# Antegrade versus retrograde facial nerve dissection in benign parotid surgery: Is there a difference in postoperative outcomes? A meta-analysis

**DOI:** 10.1371/journal.pone.0206028

**Published:** 2018-10-19

**Authors:** Mubarak Ahmed Mashrah, Taghrid Ahmed Al-dhohrah, Fahmi Ahmed Al-zubeiry, Lingjian Yan, Faez Saleh Al-Hamed, Xiaopeng Zhao, Chaobin Pan

**Affiliations:** 1 Department of Oral & Maxillofacial Surgery, Sun Yat-sen Memorial Hospital, Sun Yat-sen University, Guangzhou, China; 2 Department of Oral & Maxillofacial Surgery, Mother and Childhood Hospital, Ministry of Health, Ibb city, Yemen; 3 Department of Oral Pathology & Medicine, Faculty of Dentistry, Ibb University, Ibb city, Yemen; 4 Department of Oral & Maxillofacial Surgery, Guanghua Stomatology Hospital, Sun Yat-sen University, Guangzhou, China; 5 Faculty of Dentistry, McGill University, Montreal, Quebec, Canada; University of Brescia, ITALY

## Abstract

**Objective:**

The primary aim of this meta-analysis was to test the null hypothesis of no difference in facial nerve dysfunction in studies that compared classical antegrade facial nerve dissection (AFND) versus retrograde facial nerve dissection (RFND) during benign parotid surgery.

**Methods:**

A comprehensive search of PubMed, the Cochrane Central Register of Controlled Trials, Scopus, Google Scholar, Science Direct and relevant journals was undertaken up to June 27, 2018. Randomized controlled clinical trials (RCTs), controlled clinical trials (CCTs), and retrospective studies aimed at comparing the effect of AFND vs. RFND during parotidectomy were included. The outcome measures included facial nerve dysfunction, Frey’s syndrome, recurrence, silaocele, salivary fistula, operating time length of hospital stay, and estimated blood loss. Pooled risk ratio (RR) and weighted mean differences (MD) with 95% confidence intervals were calculated using either a fixed-effects or random-effects model.

**Results:**

Ten studies; four RCTs and five retrospective studies were included. There were 570 patients (319 in RFND group and 251 in AFND group). 481 patients in 9 studies reported the incidence rate of facial nerve dysfunction. No statistical significant difference was observed between both groups concerning the occurrence of transient or permanent facial nerve paralysis (p = 0.44 and 0.11 respectively). One out 10 studies reported the incidence rate of sialocele, however no statistical difference was observed between the two techniques. There was reduction in the operative time (19.30 min), amount of blood loss (25.08 ml) and amount of healthy salivary tissues removed (12.20 mm) in RFND compared with AFND.

**Conclusions:**

According to the results of the current review there is no evidence demonstrating a significant advantage of one approach over another, therefore, well-designed standardized RCTs are required.

## Introduction

Parotidectomy is one of the most frequently performed surgical procedures in maxillofacial and otolaryngological departments. This procedure may result in detrimental complications to the patients including: facial nerve paralysis, Frey’s syndrome, sialocele and salivary fistula [1–5].

In 1823, Bernard M[6] reported the first case of parotid tumor resection. Before the 1940s, surgical excision of parotid gland pleomorphic adenoma was associated with a considerable rate of permanent facial nerve paralysis and recurrence (20–45%) [7,8].

It was advocated by Janes [9] and Bailey[10] to identify the main trunk of the facial nerve first, followed by removal of the superficial and/or deep lobe of the parotid gland. Using this technique, the reported recurrence rate and permanent facial nerve paralysis rate become very rare, decreasingto (0.2%) and (2.2%) respectively[11].

Generally, facial nerve dissection during partial or total parotidectomy can be achieved using two anatomic basic approaches: antegrade facial nerve dissection (AFND) or retrograde facial nerve dissection (RFND). With the AFND technique, the facial nerve trunk is identified as it leaves the stylomastoid foramen and then traced to its bifurcation and peripheral branches[12]. To locate the facial nerve trunk and allow for AFND approach, a number of anatomical landmarks have been used including:tympanomastoid suture, tragal pointer, and posterior belly of the digastric muscle. However, locating the facial nerve trunk could be a challenge even for an experienced surgeon operating on an obese patient. In addition, it is difficult to locate facial nerve trunk in patients requiring revision surgeries or those with large tumors[13]. For such patients, the RFND approach is the most useful. It involves identifying the mandibular or another facial nerve branch first and then dissecting back to the main trunk. According to the studies conducted by Bhattacharyya et al[12], Yu et al[14], and Liu et al[15], retrograde approach is less time-consuming, reduces intraoperative blood loss, and reduces the normal parotid tissue being removed compared with the antegrade approach.

In a survey performed in UK, 87% of practicing oral and maxillofacial, ear, nose, and throat surgeons used the antegrade technique for parotid surgery, 4% used the retrograde technique, and 9% employed mixed techniques[16]. In another survey conducted in Nigeria, including maxillofacial and ear, nose, and throat surgeons, they found that 47.5% of surgeons routinely used the antegrade technique, 12.5% used the retrograde technique, and 40% used a combination of both techniques[17].

Despite great improvement in surgical techniques of the parotid gland, there is still a risk of postoperative complications[18]. The rate of post-parotidectomy complications depends on surgical expertise, tumor size, location, and differentiation. Furthermore, revision surgery appears to be associated with more postoperative complications[3]. In an attempt to reduce postoperative complications, intraoperative facial nerve monitoring was used. It was reported that intraoperative facial nerve monitoring reduced the incidence of postoperative facial nerve weakness to 14.6% compared to 48.5% without monitoring[19].

In a systematic review comparing the effect of total parotidectomy versus superficial parotidectomy in management of benign parotid gland tumors, they found that the rate of transitory facial nerve paresis ranged from 0% to 23% (mean 6.75%) in superficial parotidectomy, whereas it ranged from 0% to 45% (mean 15%) in total parotidectomy. However, permanent facial nerve paralysis was less common and it ranged from 0% to 3% (mean 0.8%) in superfacial parotidectomy and from 0% to 17% (mean 4.4%) in total parotidectomy[20].

Ruohoalho et al[2] stated that up to half of the patients experienced facial palsy after benign parotid surgery. They reported that immediate postoperative facial palsy rated in subgroups of partial superficial parotidectomy, superficial parotidectomy, extended parotidectomy, and extracapsular parotidectomy were 41.5%, 43.8%, 53.8%, and 6.3%, respectively. Kadletz et al[21] concluded that extracapsular dissection of benign parotid tumors led to a significantly higher percentage of permanent facial palsy, Frey’s syndrome, recurrent disease, and positive resection margins compared to superficial parotidectomy. It was reported that frequency rate of Frey’s syndrome after parotidectomy ranged from 12.5% to 62%, as assessed by subjective methods and 22% to 98% using Minor’s starch-iodine test[2,22].

To date, although a considerable number of systemic reviews comparing the surgical outcomes and complications of different parotidectomy surgical techniques have been reported in literature[11,23–28], there is no evidence summarizing the incidence of facial nerve dysfunction using RFND versus AFND approaches in managing patients with parotid gland benign lesions. This study was conducted to systematically review and critically analyze the available evidence regarding the role of RFND and AFND approaches in the frequency of facial nerve dysfunction and other complications following surgical excision of parotid gland benign tumors and inflammatory diseases.

## Methods

This systematic review was conducted in accordance with the Preferred Reporting Items for Systematic reviews and Meta-Analyses (PRISMA) statement [29]

### Search strategies

From inception until June 27, 2018, a comprehensive electronic search of the major databases (PubMed, CENTRAL (Cochrane library), GOOGLE SCHOLAR, SCOPUS and SCIENCE DIRECT) was performed independently by 2 reviewers (M. MA, A. FS). In addition, manual searching of the online databases of the top 5 journals with the highest impact factor in the field of oral and maxillofacial surgery and otolaryngology- head and neck surgery such as American Journal of Oral and Maxillofacial Surgery, International Journal of Oral and Maxillofacial Surgery, British Journal of Oral and Maxillofacial Surgery, journal of oral oncology Journal of Cranio-Maxillo-Facial Surgery, JAMA Otolaryngology-Head & Neck Surgery, CLINICAL OTOLARYNGOLOGY, HEAD AND NECK, OTOLARYNGOLOGY-HEAD AND NECK SURGERY, and Journal of Otolaryngology-Head & Neck Surgery were performed. A combination of the following key words was used for the electronic search: "Neoplasms"[Mesh]) OR "Parotid Gland"[Mesh]) OR "Salivary Glands"[Mesh] AND "parotidectomy" OR "anterograde" OR "retrograde" AND "Clinical Trial"[Publication Type]) OR "Non-Randomized Controlled Trials as Topic"[Mesh]) OR "Retrospective Studies"[Mesh])) AND "Facial Paralysis"[Mesh] OR "Frey's syndrome" OR "Salivary Gland Fistula"[Mesh] OR "complications"[Subheading]) OR "sialocele"). The reference lists of relevant articles were manually checked for studies that could meet the inclusion criteria.

### Inclusion and exclusion criteria

The inclusion criteria included all English-language randomized clinical trials (RCTs), controlled clinical trials (CCTs), and retrospective studies that compared the effect of AFND versus RFND approaches used for excision of parotid gland benign tumors and other inflammatory diseases. In addition to the above criteria, each included study must report at least one of the following outcomes of interest: 1) Transient facial nerve paresis, 2) Permanent facial nerve paralysis, 3) Frey’s syndrome, 4) Salivary fistula, 5) sialocele, 7) Operative time, 8) Volume of blood loss, 9) Amount of the healthy salivary tissue removed and/or 10) Length of hospital stay.

The exclusion criteria included other studies that reported one of the following: 1) included malignant tumors, 2) Studies with <5 patients in each group, 3) Studies with revision parotid surgery 4) review studies, meeting abstracts, case reports, case series and/or non-English articles.

### Data extraction process

Two researchers (M. MA., A. TA.) independently assessed the titles, abstracts, and full-text of the relevant studies and any controversy was resolved by discussion.

The following data were collected for each study (when available): authors, publication year, country of origin, study design, mean age, age range, number of patients, male/female ratio, tumor entity, intraoperative facial nerve monitoring, preoperative biopsy, follow-up period, and outcome variables (Table 1).

**Table 1:** Characteristics of the included studies.

Author (Year),Country	Mean Age,Age Range, y	Study design	No. of patients	M: F ratio	Tumor entity	Intraoperative facial nerve monitoring	preoperative biopsy	Follow up
O’Regan and Bharadwaj [49](2011)United Kingdom	52 y (AFND)56 y (RFND)	RCT	4020(AFND)20(RFND)	10:30	Pleomorphic adenoma, Warthin´s tumour, Sialadenitis, Sialosis and others	NR	Yes (FNA)	6 months
Abd-Elwahab et al [44](2014)Egypt	23–70 y	RCT	1610 (AFND)6 (RFND	5:11	Pleomorphic adenoma, adenolymphoma	NR	Yes (FNA)	-
Al-Na'ssan et al [45] (2008)Jordan	48.5 y (RFND)46.2 y (AFND)	RCT	4823(AFND)25 (RFND)	14:34	Benign tumors	NR	YES (FNA)	-
Scarpini et al [50](2009)Italy	45.3 y (AFND)43.8 y(RFND)	RT	6432(AFND)32(RFND)	25:39	Pleomorphic adenoma	NR	Not reported	36 to 1203–10 y
Emodi et al[46] (2009)Israel	Mean (SD) 43.8 (16.97) yRange 12-79y	RT	4818(AFND)30(RFND)	19:29	Pleomorphic Adenoma	NR	Yes (FNA)	57 months
K. Mahmood et al[48] (2010)UK	Average 58 yRange (32–84)	RT	6432(AFND)32(RFND)	31:33	Pleomorphic adenoma, Monomorphic adenoma, Adenolymphoma (Warthin´s tumour), Sialadenitis, Lymphadenitis, Granuloma, Cyst, Lipoma	Yes	Not reported	6 months
Sharma et al,[51] [49] (2011)India	average 48 yRange 27–76	RT	3929(AFND)10(RFND)	13:26	Benign tumors	Yes	Not reported	-
Li et al,[47](2014)China	Mean 40.8	RT	12958(AFND)71(RFND)	57:72	pleomorphic adenoma	NR	Not reported	62 months(range,15–98months)
Ashishkumar MB et al, (2018) [52] India	AFND mean = 38.21RFND mean = 40.72	RCT	3214(AFND)18(RFND)	34.38:65.63	pleomorphic adenoma	NR	Yes	6 months
Herbert and Morton [53](2011)New Zealand	49 y	RT	9010275 (RFND)15 (AFND)12 (Mixed)	44:58	Benign tumors	NR	Not reported	2 weeks

AFND = anterograde facial nerve dissection, RFND = anterograde facial nerve dissection, RCT = randomized controlled trial, RT = retrospective study, NR = not reported

Two researchers (M.MA. A. FA.) independently reviewed the included articles and collected the data. Disagreements between the reviewers were resolved by consensus.

### Methodological quality appraisal

Two authors (M. MA. and A TA.) independently assessed the risk of bias in the included studies.

Quality assessment was dependent on combining the proposed criteria of the Meta-Analysis of Observational Studies in Epidemiology (MOOSE) statement[30], the Strengthening the Reporting of Observational Studies in Epidemiology (STROBE) statement[31], and the Preferred Reporting Items for Systematic Reviews and Meta-Analyses (PRISMA) statement[29]. Each study was evaluated based on the following criteria: (1) random selection in the population, (2) definition of inclusion and exclusion criteria, (3) report of losses to follow-up, (4) validated measurements, and (5) statistical analysis. Any study that fulfilled all of the criteria mentioned above was classified as having a low risk of bias. Studies that did not meet one of these criteria were classified as having a moderate risk of bias. When two or more criteria were missing, the study was considered to have a high risk of bias (Table 2).

**Table 2:** Results of the quality assessment.

Author (Year), Country	Year published	Random selection in population	Defined inclusion/ exclusion criteria	Loss of follow-up	Validated measurement	Statistical analysis	Estimated potential risk of bias
O’Regan and Bharadwaj [49] (2011) United Kingdom	2011	Yes	Yes	Yes	Yes	Yes	Low
Abd-Elwahab et al [44](2014) Egypt	2014	Yes	Yes	Yes	Yes	Yes	Low
Al-Na'ssan et al [45](2008) Jordan	2008	Yes	Yes	Yes	Yes	Yes	Low
Scarpini et al [50](2009) Italy	2009	No	Yes	Yes	Yes	Yes	Moderate
Emodi et al[46](2009) Israel	2009	No	Yes	Yes	Yes	Yes	Moderate
K. Mahmood et al[48](2010) UK	2010	No	Yes	Yes	Yes	Yes	Moderate
Sharma et al, [51] (2011) India	2011	No	Yes	Yes	Yes	Yes	Moderate
Li et al,[47](2014) China	2014	No	Yes	Yes	Yes	Yes	Moderate
Ashishkumar MB et al, 2018 [52]	2018	Yes	Yes	Yes	Yes	Yes	Low
Herbert and Morton [53](2011) New Zealand	2011	No	Yes	Yes	Yes	Yes	Moderate

### Statistical analysis

In case of dichotomous data, meta-analysis of selected studies with a risk ratio (RR) comparing postoperative outcomes of AFND and RFND approaches using the Mantel-Haenszel (MH) test with corresponding 95% confidence interval (95% CI) was conducted. In case of continuous data, weighted mean differences (MD) and 95% CI were used to construct forest plots of selected studies. The heterogeneity across studies was detected by Cochrane Q test (χ2 test) and I-squared index (I^2^). I ^2^ = 0% to 25%, no heterogeneity; I ^2^ = 25% to 50%, moderate heterogeneity; I ^2^ = 50% to 75%, high heterogeneity; I ^2^ = 75% to 100%, extreme heterogeneity[32]. We used the random effect model described by DerSimonian and Laird[33] when I^2^< 50%. Otherwise, the data was regarded as homogeneous and a fixed effect model was used. P value of <0.05 was considered statistically significant. The Cochrane Collaboration’s Review Manager Software (RevMan version 5.0) was utilized for data analysis. The Begger’s test and Egger’s linear regression using STATA 12.0 software (Stata Co., College Station, TX) were also used in data analysis.

### Sensitivity analysis

If there were sufficient included studies, a sensitivity analysis was performed to assess the robustness of our meta-analysis. This was achieved by exclusion of retrospective studies with a high risk of bias.

## Results

**Fig 1:**
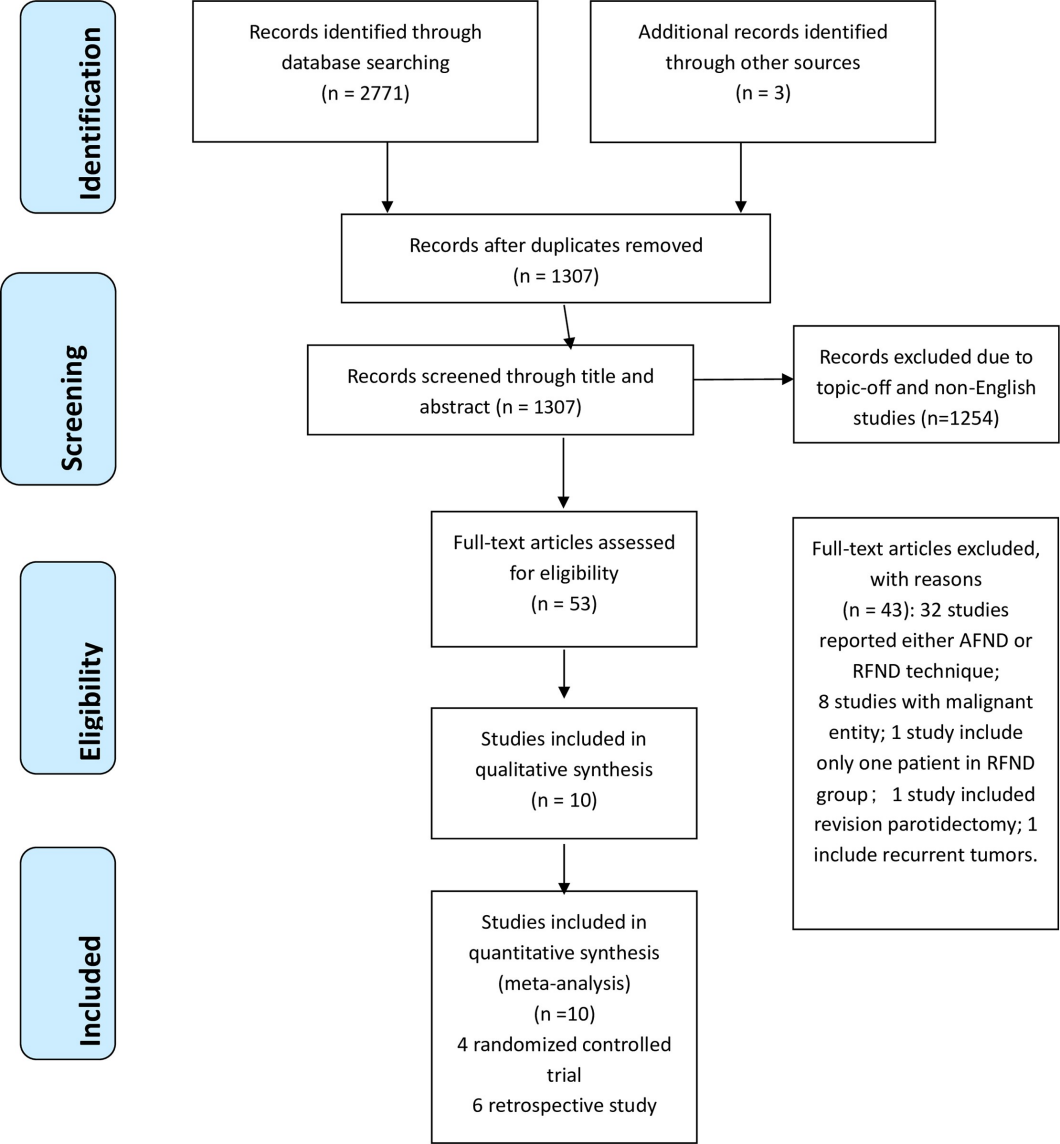
Study flow diagram.

The electronic and manual searches identified 2771 articles, 1307 records remained after duplicates were removed. The titles and abstracts of the remaining 1307 articles were screened and 1254 were excluded due to being off topic or non-English studies. The remaining 53 studies were carefully read by two researchers for potential inclusion. Of 53 full-text studies reviewed for potential inclusion; only 10 met the inclusion criteria and were assessed for reliability (Table 1). The other 43 articles were excluded with reasons (8 studies included malignant tumors[12,34–40], 1 study included only one patient in RFND group[41], 1 study included revision parotidectomy[42], 1 included recurrent tumors[43] (Table 3, Fig 1), 32 studies reported either AFND or RFND technique.

**Table 3:** Summary of the excluded studies and reason(s) for exclusion.

Study	Reason (s) for exclusion
Bhattacharyya et al (2004)[12], Chow et al (2011)[35], Anjum et al (2008)[34], Wang et al(2009)[40], Sungur et al(2002)[39], Furusaka et al(2013)[36]^,^ Shrestha et al(2011)[38](Shrestha & Gurung 2011)^,^ and Mokhtari N, et al(2010)[37]	Included both malignant and benign tumors.
Sharma and Sirohi (2010)[41]	Only one patient was in RFND group.
Guntinas-Lichius et al (2006)[42]	Included 39 patients with revision surgery.
Musani et al (2014)[43]	Included recurrent benign tumors.

**Fig 2:**
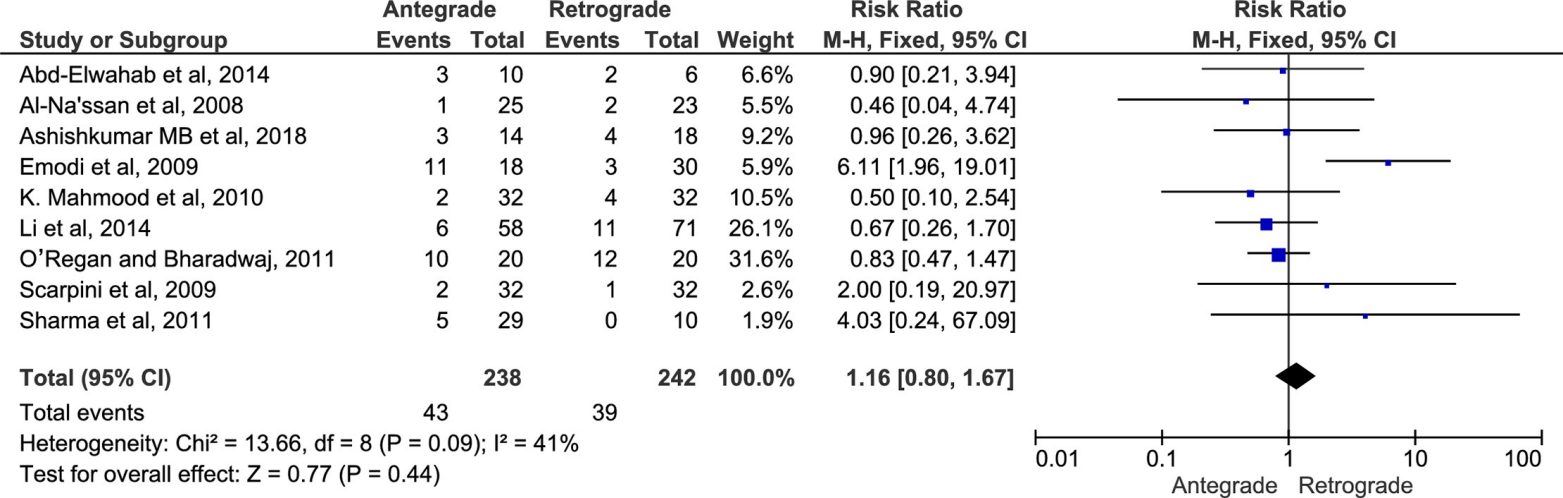
Forest plot of transient facial nerve paresis.

**Fig 3:**
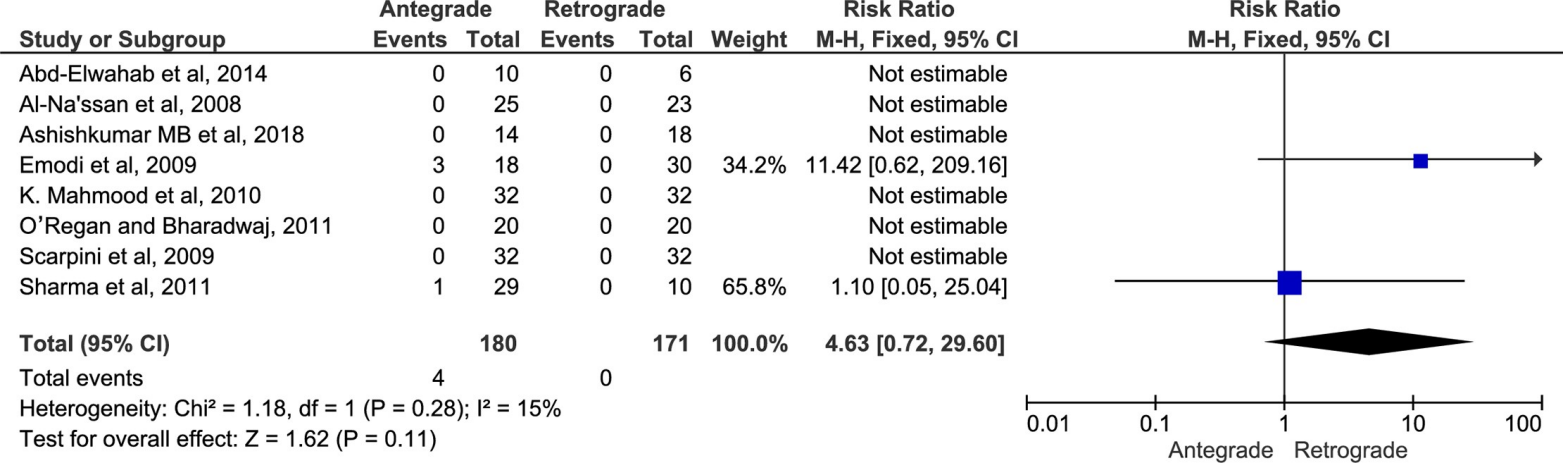
Forest plot of permanent facial nerve paralysis.

The 10 included articles had a total of 570 participants, of them 319 patients underwent RFND and 251 underwent AFND and patients age ranged from 12 to 79 years. The follow-up time ranged from two weeks to 10 years. Four studies were prospective randomized controlled trials and six studies were retrospective studies. Nine studies[44–52] evaluated facial nerve dysfunction using AFND and RFND approaches [44–46,48–52] (Figs 2 and 3).

Assessment of other variables such as Frey’s syndrome[46,47] salivary fistula,[44] [47] sialocele [53], recurrence rate[46][47] and amount of healthy salivary gland removed, [46] operative time,[45–47,52] blood loss[45,47,51] and length of postoperative stay in the hospital[39] was summarized in the Table 4 and Data underlying the meta-analysis (**S1 Data**).

**Table 4:** Results of the secondary outcomes of RFND versus AFND in this meta-analysis.

Outcome Variable	No. of patients included	No. of patients in each group	Incidence rate(%)	No. of trials	Heterogeneity test		Pooled effect	
					P(Q-test)	I-square	Effect size (95% CI)	P value
Frey’s syndrome	177	AFND = 76RFND = 101	AFND = 10.53RFND = 30.7	2	0.09	64%	Random; RR, 0.4 (0.12–1.35)	0.14
Recurrence rate	177	AFND = 76RFND = 101	AFND = 5.26RFND = 2.97	2	0.95	0	Fixed; RR, 1.83(0.40–8.48)	0.44
Sialocele	90	AFND = 15RFND = 75	AFND = 20RFND = 21.33	1	-	-	Fixed; RR, 0.92(0.23–3.67)	0.91
Salivary fistula	154	AFND = 77RFND = 77	AFND = 10.39RFND = 10.39	2	0.13	56%	Random;RR, 0.63(0.06–6.17)	0.69
Blood loss	216	AFND = 112RFND = 104	-	3	<0.00001	99%	Random; MD, 25.08 ml (-12.06–62.22)	0.19
Operative time	225	AFND = 99RFND = 126	-	3	0.12	53%	Random; MD, 10.63 min (-1.80–25.28)	0.09
Normal salivary tissue removed	48	AFND = 18RFND = 30	-	1	-	-	Fixed; MD, 12.20 mm (4.63–19.77)	0.002
Length of postoperative stay in the hospital	129	AFND = 58RFND = 71	-	1	-	-	Fixed; MD, 0.1 day (-0.32–0.52)	0.64

### Quality assessment and publication bias

**Fig 4:**
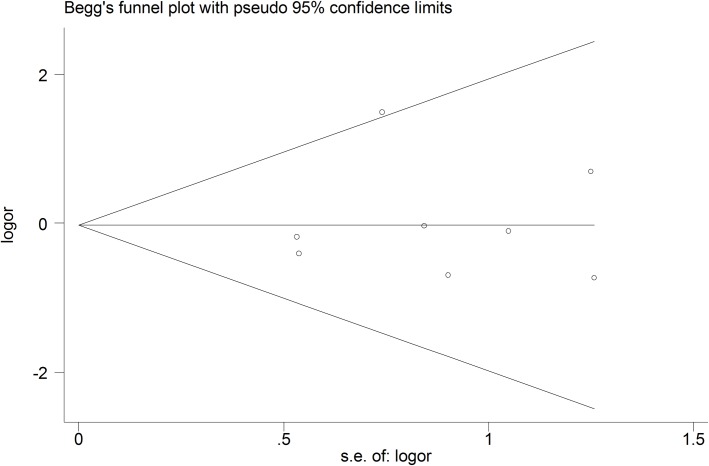
Begg’s funnel plot for transient facial nerve paresis.

**Fig 5:**
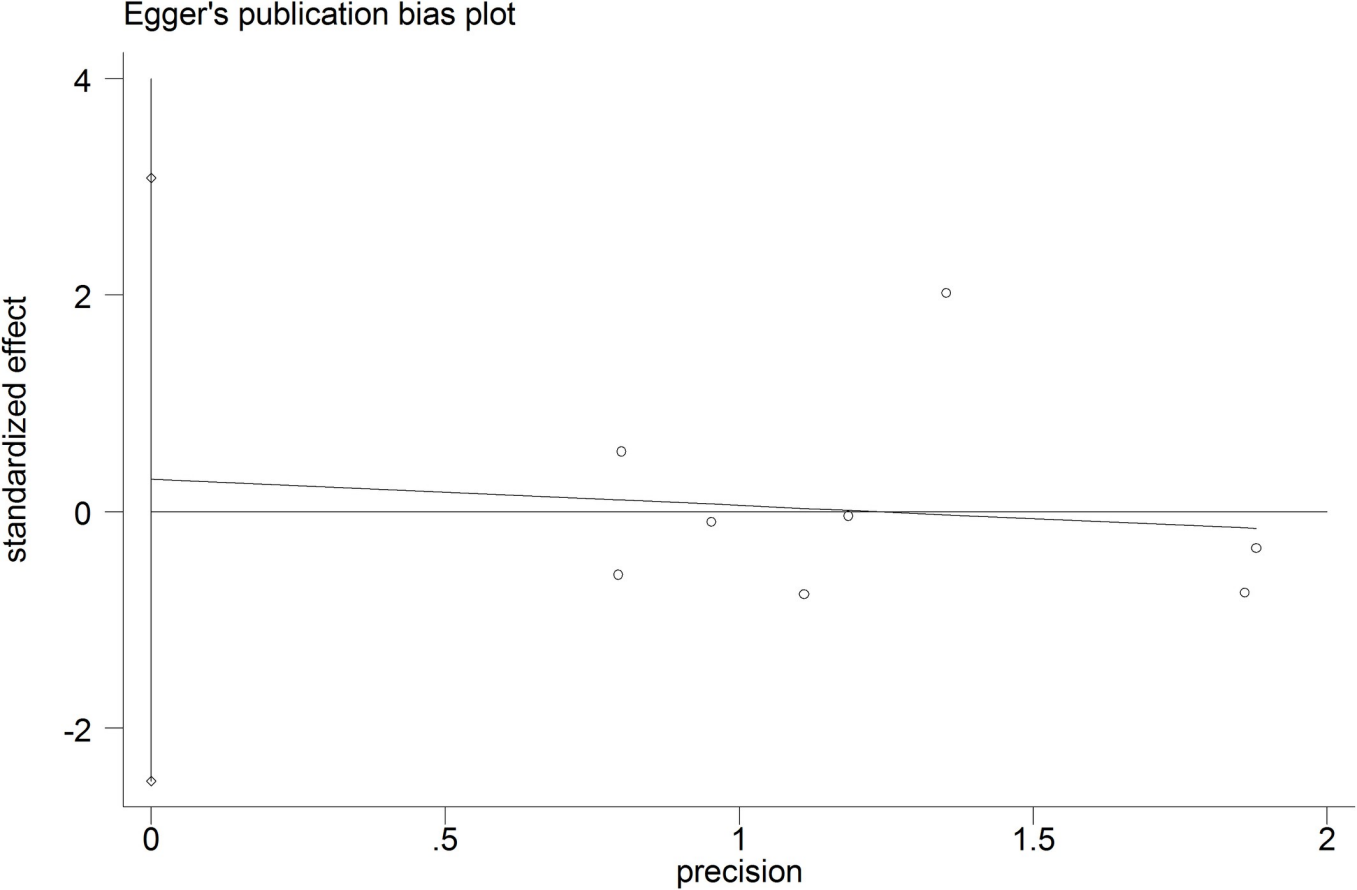
Egger’s linear regression for transient facial nerve paresis.

Concerning quality assessment of included studies, four studies were considered as having low risk of bias[44,45,49,52], and six studies were scored as moderate risk of bias[46–48,50,51,53]. (Table 2). Begg’s test and Egger’s was performed when the number of the included studies was more than 3. No publication bias was observed in both Begg’s test and Egger’s linear regression (P = 0.805) and (P = 0.803) respectively. (Figs 4 and 5)

### Results of individual outcome variables

A total of 481 participants enrolled in 9 studies[44–52] compared AFND (n = 243) vs RFND (n = 238) regarding facial nerve dysfunction. The incidence of transient facial nerve weakness was 16.4% in AFND and 16.04% in RFND and no statistical difference was observed (Fixed; RR, 1.16; 95% CI, 0.80–1.67, P = 0.44, *I*^*2*^ = 41%). The incidence of permanent facial paralysis was 2.41% in AFND and 0% in RFND and no statistical difference was observed (Fixed; RR, 4.63; 95% CI, 0.72–29.04, P = 0.11, *I*^*2*^ = 15%) (Figs 2 and 3). Frey’s syndrome was evaluated only in 2 studies[46,47] with total of 177 patients (AFND = 76 vs. RFND = 101). The incidence rate was 10.5% and 30.7 in AFND and RFND respectively. However, the pooled effect showed no statistical difference between the two approaches (Random, RR, 0.40; 95% CI, 0.12–1.35, P = 0.14, *I*^*2*^ = 64%). Furthermore, there was no statistical difference between the two approaches regarding recurrence rate, and salivary fistula (P = 0.44 and 0.69 respectively) (Table 4). Operative time was evaluated in three studies[46,47,52] with 209 patients (90 AFND and 119 RFND). There was a 19.30 min reduction in RFND compared with AFND. The pooled overall effect was (Random; MD, 19.30 min; 95% CI, -2.79–41.39, P = 0.09). The volume of blood loss during surgery was reported in three studies[45,47,51]. Two studies[45][51] reported significant reduction in volume of blood loss in RFND versus AFND whereas one study[47] reported non-significant difference in volume of blood loss between both groups (Table 4). Sialocele [53] and the length of postoperative hospital stay[47] were evaluated only in two separate studies. There was insignificant difference between both groups regarding the incidence of sialocele and the length of postoperative hospital stay (P = 0.810, and 0.516 respectively). Only one study[46] evaluated the amount of excised healthy parotid tissue. There was a significant amount of excised healthy parotid tissue in AFND versus RFND approaches (P = 0.01).

### Sensitivity analysis

Exclusion of the retrospective studies didn’t change the overall results.

## Discussion

Whatever the type of parotidectomy surgical technique performed, dissection and preservation of the facial nerve can only be achieved using two approaches; antegrade or retrograde. To the best of our knowledge, this is the first systemic review and meta-analysis that compared the AFND and RFND approaches used in parotidectomy regarding the incidence of facial nerve paralysis and other complications in benign parotid surgery. The findings of this review concluded that there is no significant difference between AFND and RFND approaches in term of transient facial nerve paresis (Fixed; RR, 1.16; 95% CI, 0.80–1.67, P = 0.44, *I*^*2*^ = 41%), permanent facial nerve paralysis (Fixed; RR, 4.63; 95% CI, 0.72–29.04, P = 0.11), recurrence rate, Frey’s syndrome, salivary fistula and sialocele.

Our findings are consistent with the studies conducted by Abd-Elwahab et al,[44] O’Regan and Bharadwaj[49], Mahmood et al,[48] and Guntinas-Lichius et al[42] in which no statistical difference was observed between AFND and RFND in regarding of transient and permanent facial nerve injury. In contrast, our results were inconsistent with the studies conducted by Emodi et al[46] and, Furusaka et al[36] in which the incidence of facial nerve paralysis was significantly higher in the anterograde parotidectomy group compared to the retrograde parotidectomy group (p < 0.05).

In a recent systemic review and meta-analysis conducted by Stankovic et al, [54] the authors concluded that no significant difference was observed between AFND and RFND in regards to facial nerve dysfunction. This finding was in line with our finding, however, the authors included three studies which were excluded from our systemic review and meta-analysis due to inclusion of malignant tumors [12,34,36](Table 3). Additionally, they did not include four more studies with benign tumors in their meta-analysis[44,45,47,51]. Moreover, the current systemic review and meta-analysis also reported the outcomes of AFND versus RFND in regard to Frey’s syndrome, recurrence rate, sialocele, salivary fistula and blood loss which were not reported in the Stankovic et al’s study[54] (Table 3) and (**S1 Data**).

There was a reduction in the operative time (19.30 min), and blood loss (25.08 ml) in RFND compared with AFND. (Table 4) However, P value was not statistically significant (0.09 and 0.19 respectively), which was inconsistent with the studies conducted by Bhattacharyya et al,[12] Chow et al[35] and Wang et al[40].

Several factors have been considered as the potential risk factors of post-parotidectomy complications (facial nerve deficit, Frey’s syndrome, salivary fistula and recurrence rate)[3,19,55]. These included the extent of surgery, the tumor entity, the size of the lesion and the surgeon’s experience. The current review suggests that the method of identification and dissection of the facial nerve may also be considered as a risk factor for post-parotidectomy complications. However, our findings showed no statistical difference between AFND and RFND regarding the above-mentioned complications.

Cannon et al [56]concluded that the greater the length of facial nerve dissected, the higher the chance of facial nerve paresis. Although the AFND associated with greater length of facial nerve dissection compared with RFND, no statistical difference was observed between the two approaches concerning the above-mentioned complications.

A thorough knowledge of the physiopathological mechanisms of post-surgical nerve weakness is still poorly understood. However, there are several possible aetiologies that may cause facial nerve dysfunction after parotid surgery; including trauma (compression, crushing, and stretching), heat damage from electro-coagulators, damage from overzealous nerve stimulation and edema in the surgical area. Such etiologies normally require 6 to 12 month to recover in case of temporary nerve injury [57].

Some risk factors for facial nerve weakness after parotidectomy have been considered in the literature,[58] such as pathological entity, age, gender, and secondary surgery. A recent review of 334 patients conducted by Maddox et al, [59] they reported that malignant tumors have been considered as a risk factor associated with a higher incidence of postoperative nerve dysfunction. The malignant parotid tumors characterized by infiltrating of the neighboring structures such as facial nerve and skin, and metastasized to the neck lymph nodes. Therefore, a multidisciplinary approach in the management of malignant tumors of the parotid tumors is needed, this may include total parotidectomy, neck dissection and/or adjuvant radiochemotherapy.[60]

Owusu et al[58] concluded that age, gender, and pathologic diagnosis were not predictive of postoperative nerve dysfunction. Owusu et al also concluded that the risk of facial nerve injury in pediatric patients is comparable to that of the adult population. In one of the largest study (n = 75 cases) analyzing the clinical outcome of patients treated surgically for chronic parotid sialadenitis, Patel et al, stated that the incidence of temporary and permanent postoperative facial nerve neuropraxia is similar to the incidence reported in benign parotid tumor surgery[61]. In regards to a revision surgery, the incidence rates of temporary and permanent facial nerve dysfunction are considerably higher after revision parotid surgery and are reported to be 90% to 100% and 11.3% to 40.0%, respectively. [62]

In the current review, from 9 included studies, only two retrospective studies [46,51] reported the incidence of permanent facial nerve paralysis in the AFND group. The reported incidence rate of permanent facial nerve paralysis was 2.41% in the AFND approaches and 0% in the RFND, however, no statistical difference was observed between the two techniques.

A total of 177 patients in two included studies[46,47]evaluated the incidence of Frey’s syndrome, we found that no statistical difference existed between AFND technique and RFND technique (P = 0.14). This also was in agreement with the study performed by Chow et al[35] which was excluded from our review (Table 2).

A number of clinical studies suggested that the amount of glandular tissue removed during parotidectomy is one of the most important risk factors for the development of Frey’s syndrome[25], the recurrence rate[21] and sialocele/ salivary fistula[63]. Although in the present review the amount of salivary tissue excised during the antegrade parotidectomy was significantly greater than that of retrograde parotidectomy (P = 0.002); however, no statistical difference in the incidence rate of Frey’s syndrome was observed between the two approaches. Moreover, no statistical difference was observed between the two approaches in regard to the rate of recurrence. This conclusion depends on the outcomes obtained from two studies with 177 patients, so it could not be used as a reference and further trials are recommended.

One study evaluated the incidence rate of sialocele [53] and two studies reported the incidence of salivary fistula[44,47], however, no statistical difference was reported between AFND and RFND parotidectomies. In a study performed by Britt et al,[64] they reported that no association has been observed between the volume of tissue removed during parotidectomy and the incidence rate of sialocele or salivary fistula and this was in line with our findings.

Several limitations have been noticed for the current systemic review and meta-analysis. First, it did not include non-English studies. Second, most of studies included in this review, were retrospective except four studies and they focused mostly on single variable (facial nerve weakness), and ignored the other variables.

Third, House-Brackmann and Sunnybrook scales were not consistently used for grading of facial paresis. Four, the incidence of permanent facial nerve paralysis was only reported in two retrospective studies. So, in the absence of randomization, the selection of surgical approach was often based on the surgeon’s preference which might have biased outcomes. Five, a few studies with small sample size evaluated the incidence of Frey’s syndrome, recurrence rate, sialocele, salivary fistula, operative time, blood loss, and amount of normal salivary tissues removed. Therefore, this meta-analysis may have not been adequately powered to detect the true difference between the two interventions.

In conclusion, AFND and RFND can be performed successfully with the same surgical outcomes. AFND is the established technique, however, RFND is the most useful if direct identification of the nerve trunk is difficult, and in revision procedures. Considering the availability of only two approaches used to dissect facial nerve during parotidectomy, there is still insufficient evidence regarding which dissection approach produces the best results in the treatment of parotid tumors. Therefore, future prospective well designed RCT with large sample size and long follow-up are recommended.

## Supporting information

S1 Checklist PRISMA 2009 checklist in this meta-analysis. (DOCX)

S1 Data Data underlying the meta-analysis. (PDF)
