# Correction: Antegrade versus retrograde facial nerve dissection in benign parotid surgery: Is there a difference in postoperative outcomes? A meta-analysis

**DOI:** 10.1371/journal.pone.0242299

**Published:** 2020-11-10

**Authors:** Mubarak Ahmed Mashrah, Taghrid Ahmed Al-dhohrah, Fahmi Ahmed Al-zubeiry, Lingjian Yan, Faez Saleh Al-Hamed, Xiaopeng Zhao, Chaobin Pan

In the Funding section, the grant number from the funder Foundation of Guangzhou Science and Technology Bureau is listed incorrectly. The correct grant number is: 201704020130.
