# Supplementary material for: Antegrade versus retrograde facial nerve dissection in benign parotid surgery: Is there a difference in postoperative outcomes? A meta-analysis
Source: PLoS One. 2018 Oct 19;13(10):e0206028. doi: 10.1371/journal.pone.0206028 (PMC6195282; doi:10.1371/journal.pone.0206028)

Antegrade versus retrograde facial nerve dissection in benign parotid surgery: is there a difference in postoperative outcome? A meta-analysis

Secondary outcomes

Forest plot of Frey’s syndrom

	Antegrade		Retrograde			Risk Ratio
Study or Subgroup	Events	Total	Events	Total	Weight	M-H, Random, 95% CI
Emodi et al, 2009	4	18	9	30	49.7%	0.74 [0.27, 2.06]
Li et al, 2014	4	58	22	71	50.3%	0.22 [0.08, 0.61]
**Total (95% CI)**		**76**		**101**	**100.0%**	**0.40 [0.12, 1.35]**
Total events	8		31			

Heterogeneity: Tau² = 0.49; Chi² = 2.82, df = 1 (P = 0.09); I² = 64%
Test for overall effect: Z = 1.47 (P = 0.14)

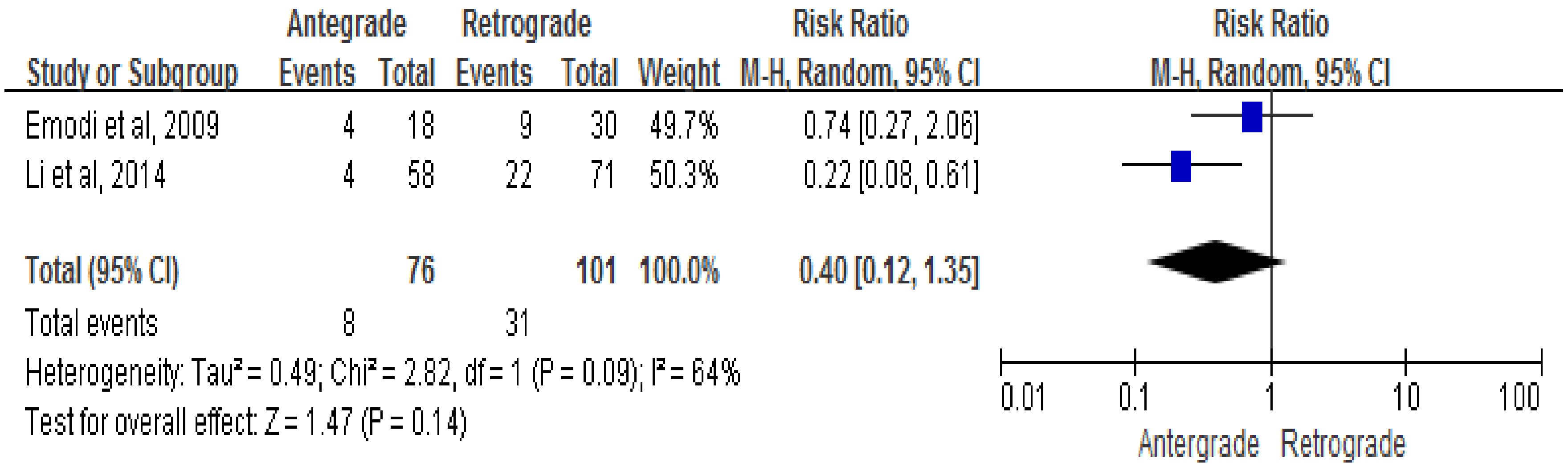

Forest plot of Sialocele

Study or Subgroup	Antegrade Events	Antegrade Total	Retrograde Events	Retrograde Total	Weight	Odds Ratio M-H, Fixed, 95% CI
Herbert and Morton, 2011	3	15	16	75	100.0%	0.92 [0.23, 3.67]
**Total (95% CI)**		**15**		**75**	**100.0%**	**0.92 [0.23, 3.67]**
Total events	3		16			

Heterogeneity: Not applicable

Test for overall effect: Z = 0.12 (P = 0.91)

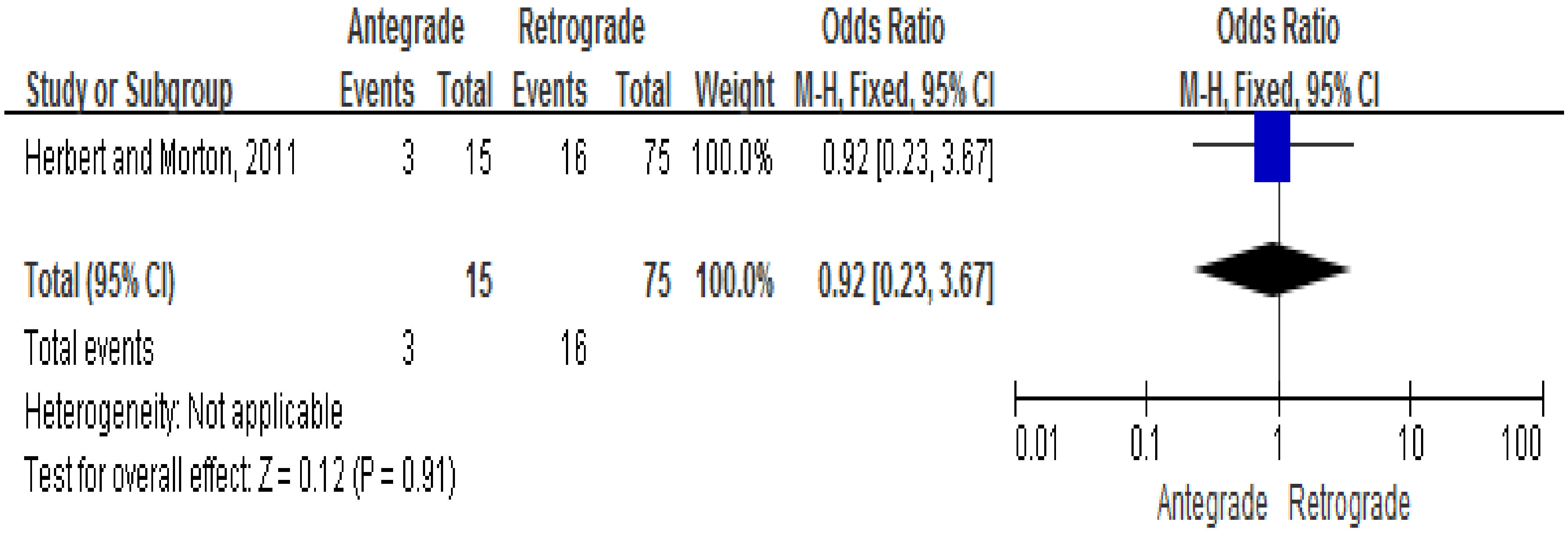

Forest plot of Blood loss

Study or Subgroup	Antegrade Mean	Antegrade SD	Antegrade Total	Retrograde Mean	Retrograde SD	Retrograde Total	Weight	Mean Difference IV, Random, 95% CI
Al-Na'ssan et al, 2008	68	4.14	29	25.4	1.4	10	35.9%	42.60 [40.86, 44.34]
Li et al, 2014	45.4	14.5	58	48.2	14.5	71	35.7%	-2.80 [-7.83, 2.23]
Sharma et al, 2011	88	56.73	25	50	56.73	23	28.3%	38.00 [5.87, 70.13]
**Total (95% CI)**			**112**			**104**	**100.0%**	**25.08 [-12.06, 62.22]**

Heterogeneity: $Tau^2 = 998.48$; $Chi^2 = 279.54$, $df = 2$ ($P < 0.00001$); $I^2 = 99\%$

Test for overall effect: $Z = 1.32$ ($P = 0.19$)

Mean Difference
IV, Random, 95% CI

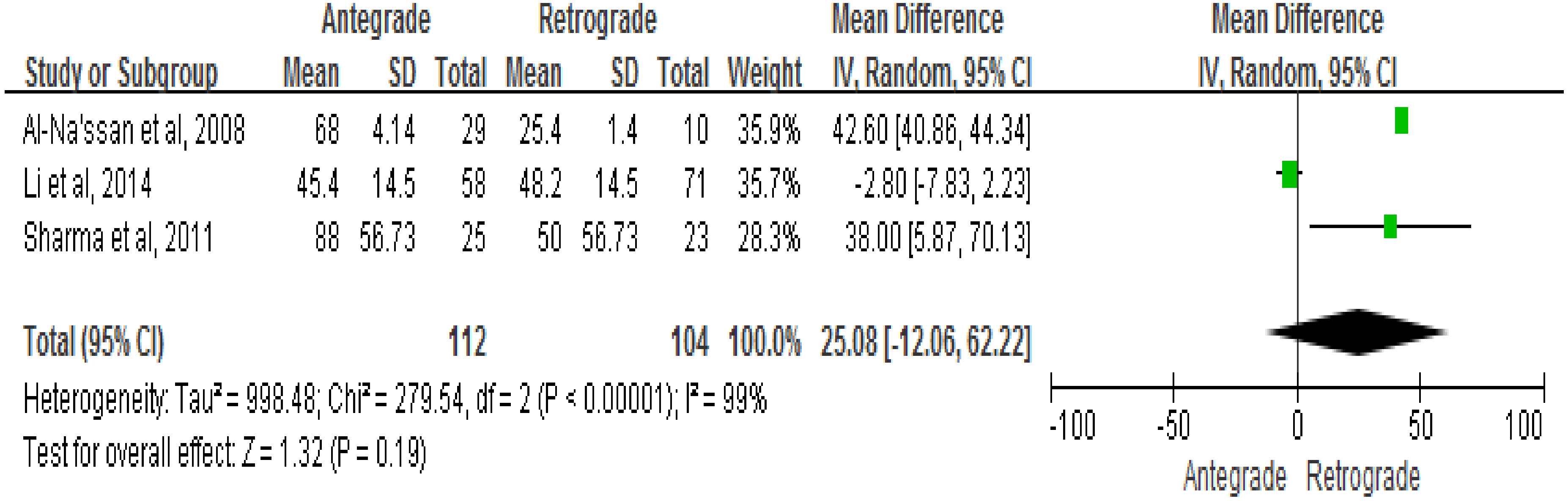

Forest plot of Salivary fistula

	Antegrade		Retrograde			Odds Ratio
Study or Subgroup	Events	Total	Events	Total	Weight	M-H, Random, 95% CI
Abd-Elwahab et al, 2014	0	19	1	6	32.8%	0.09 [0.00, 2.65]
Li et al, 2014	8	58	7	71	67.2%	1.46 [0.50, 4.31]
**Total (95% CI)**		**77**		**77**	**100.0%**	**0.59 [0.05, 7.47]**
Total events	8		8			

Heterogeneity: Tau² = 2.18; Chi² = 2.36, df = 1 (P = 0.12); I² = 58%

Test for overall effect: Z = 0.40 (P = 0.69)

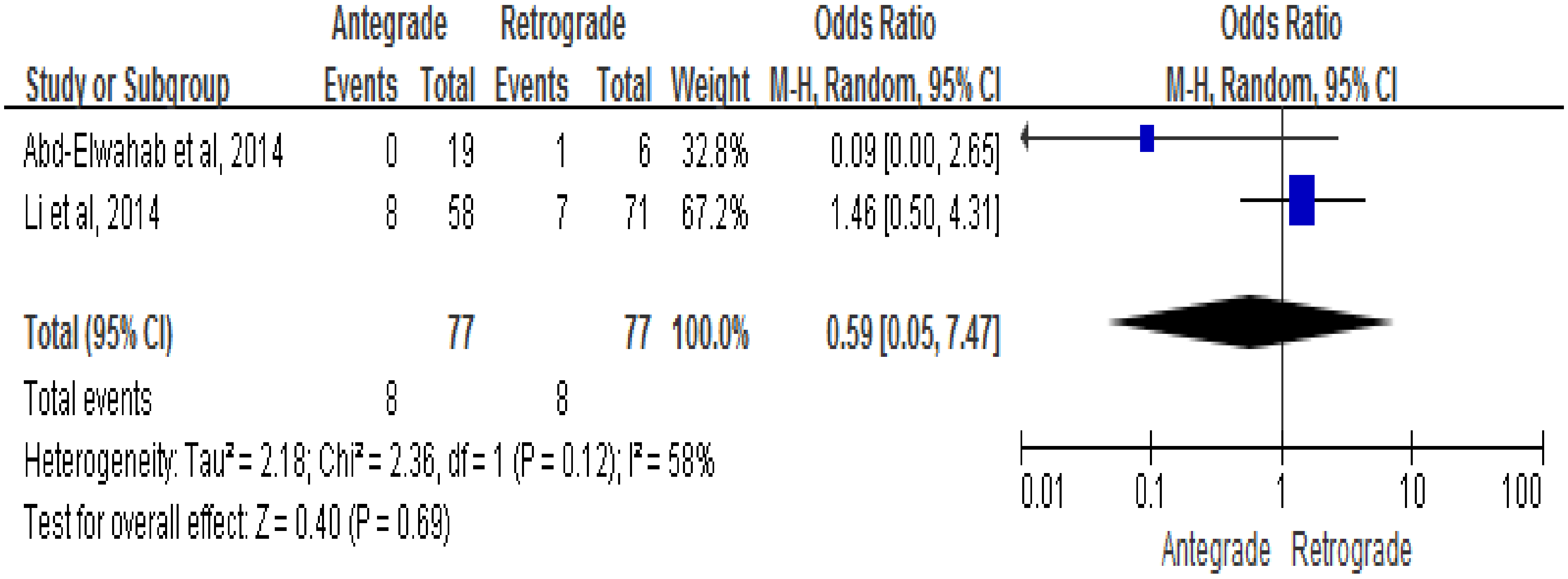

Forest plot of Operative time

Study or Subgroup	Antegrade Mean	Antegrade SD	Antegrade Total	Retrograde Mean	Retrograde SD	Retrograde Total	Weight	Mean Difference IV, Random, 95% CI
Ashishkumar MB et al, 2018	134.64	7.96	14	103.88	10.08	18	38.1%	30.76 [24.51, 37.01]
Emodi et al, 2009	171	49.7	18	145	42.7	30	24.4%	26.00 [-1.58, 53.58]
Li et al, 2014	86	20.9	58	82.7	23.7	71	37.5%	3.30 [-4.40, 11.00]
**Total (95% CI)**			**90**			**119**	**100.0%**	**19.30 [-2.79, 41.38]**

Heterogeneity: $Tau^2 = 322.96$; $Chi^2 = 29.63$, df = 2 ($P < 0.00001$); $I^2 = 93\%$

Test for overall effect: Z = 1.71 (P = 0.09)

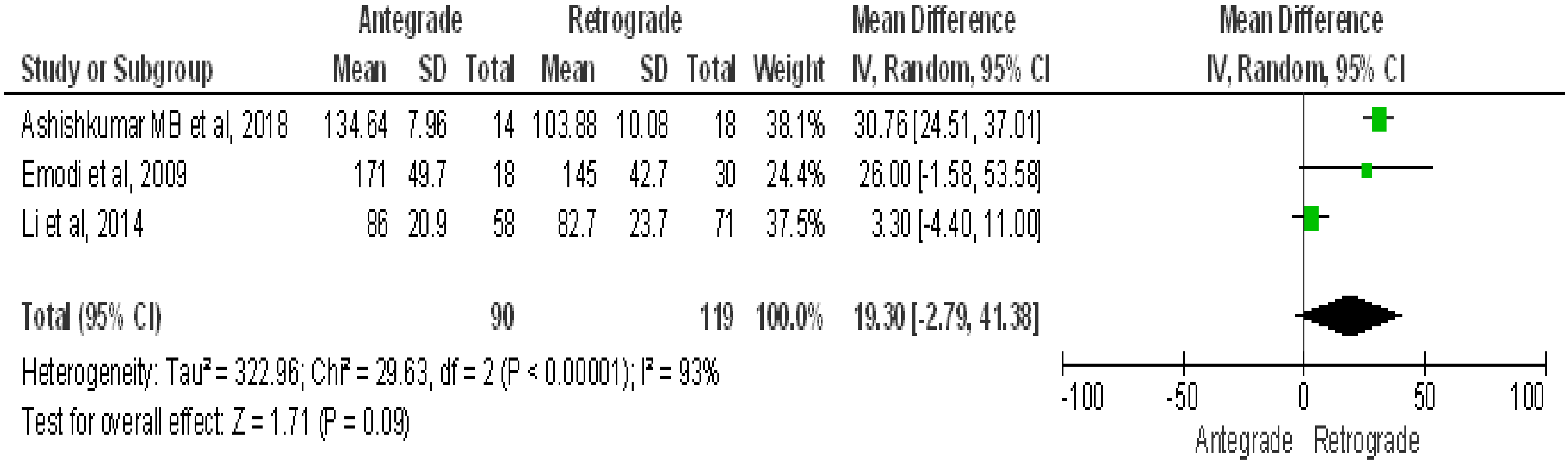

Forest plot of Length of hospital stay

	Antegrade			Retrograde				Mean Difference
Study or Subgroup	Mean	SD	Total	Mean	SD	Total	Weight	IV, Fixed, 95% CI
Li et al, 2014	5.5	1.2	58	5.4	1.2	71	100.0%	0.10 [-0.32, 0.52]
**Total (95% CI)**			**58**			**71**	**100.0%**	**0.10 [-0.32, 0.52]**

Heterogeneity: Not applicable

Test for overall effect: Z = 0.47 (P = 0.64)

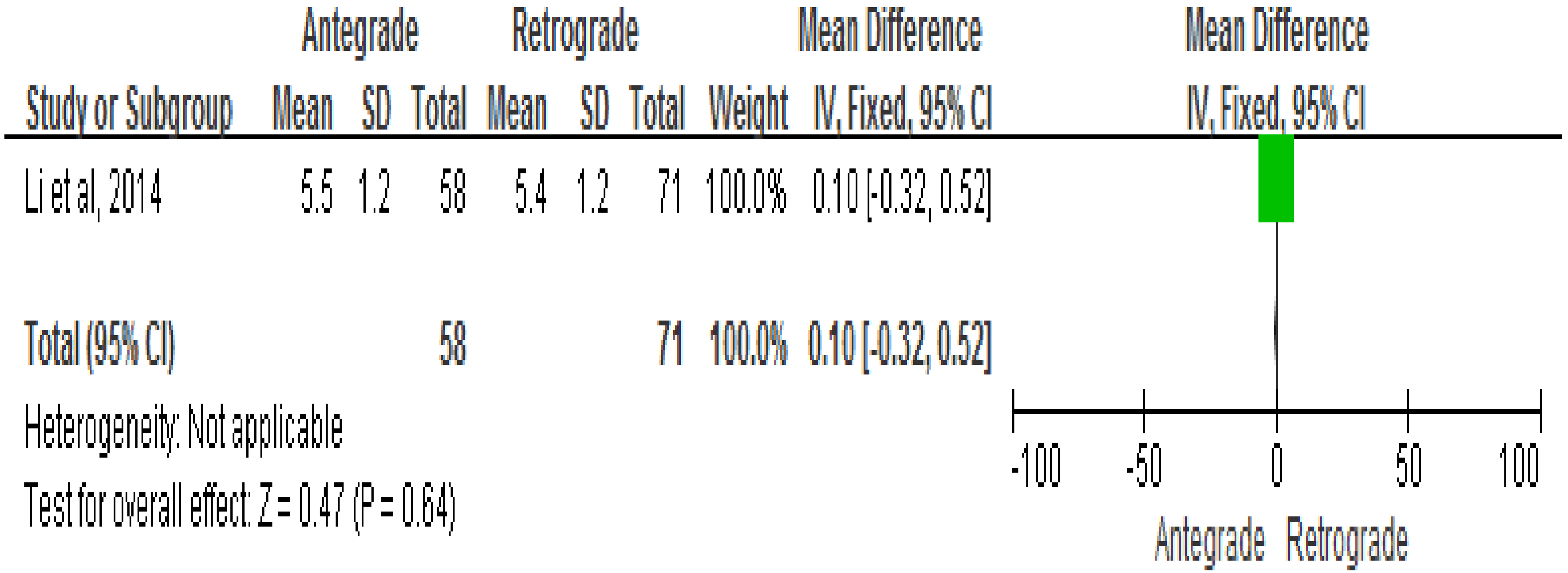

Forest plot of recurrence

Study or Subgroup	Antegrade Events	Antegrade Total	Retrograde Events	Retrograde Total	Weight	Odds Ratio M-H, Fixed, 95% CI
Emodi et al, 2009	1	18	1	30	29.3%	1.71 [0.10, 29.07]
Li et al, 2014	3	58	2	71	70.7%	1.88 [0.30, 11.66]
**Total (95% CI)**		**76**		**101**	**100.0%**	**1.83 [0.40, 8.48]**
Total events	4		3			

Heterogeneity: Chi² = 0.00, df = 1 (P = 0.95); I² = 0%
Test for overall effect: Z = 0.77 (P = 0.44)

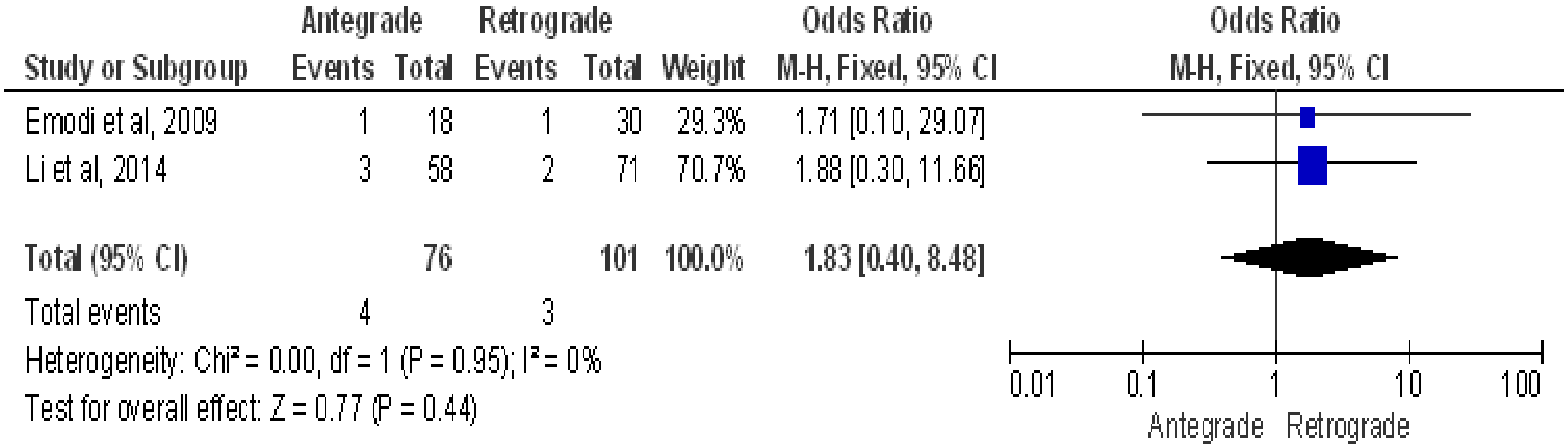

Supplement: S1 Data — (PDF) [file pone.0206028.s002.pdf]
